# Primary Cardiac Synovial Sarcoma: A Case Report

**Published:** 2017-01

**Authors:** Mahmood Hosseinzadeh Maleki, Moein Aboobakri Makouei, Farbod Hatami, Reza Zeinabadi Noghabi

**Affiliations:** *Atherosclerosis and Coronary Artery Research Center, Birjand University of Medical Sciences, Birjand, Iran. *

**Keywords:** *Heart neoplasms*, *Sarcoma*, *Echocardiography*, *Cardiac surgical procedures*

## Abstract

Primary cardiac sarcomas are rare clinical entities with an incidence rate of 0.0001% in collected autopsy series and are regarded as very aggressive tumors. We herein describe a 21-year-old woman presenting with syncope, dyspnea, and abdominal distention. She suffered from massive ascites, plural effusion, and liver congestion demonstrated by abdominal sonography and chest X-ray. Transthoracic echocardiography revealed a heterogeneous solid mass located in the right atrium; therefore, the patient underwent radical surgical excision of the tumor and 3 cycles of adjuvant chemotherapy. Fifteen months after surgery, she was having a favorable life quality without any evidence of recurrence.

## Introduction

Primary cardiac sarcomas are rare clinical entities with an incidence rate of 0.0001% in collected autopsy series and are considered as very aggressive tumors and rare malignancies, comprising approximately 5% of cardiac sarcomas and 1% of all primary cardiac tumors.^1^^, ^^2^ The majority of the literature describes a uniformly dismal prognosis with a median survival of only 6 months for these aggressive tumors. Standard surgery, adjuvant chemotherapy, and radiotherapy have been consistently unsuccessful.^3^ These sarcomas constitute the main malignant primary heart tumors with higher prevalence in men between the third and fourth decades of life and are predominantly located in the right atrium. As benign lesions, the clinical presentation of malignant cardiac tumors also depends on the location and not their histological type. ^4^

## Case Report

A 21-year-old woman presented with dyspnea and weakness. The symptoms were initially managed as the common cold. However, with the progression of the symptoms and addition of abdominal distention and syncope to the condition, she underwent abdominal sonography, which showed massive ascites, pleural effusion, and liver congestion. Chest X-ray confirmed bilateral pleural effusion, but there no evidence of abnormality in limb X-ray. Transthoracic echocardiography (TTE) revealed a large heterogeneous solid mass, measuring 7.5 × 5.5 × 3.2 cm, located in the right atrium and protruding into the pulmonary artery causing pulmonary artery obstruction. A metastatic workup, including computed tomography (CT) scan of the chest, abdomen, and pelvis, as well as a laboratory workup disclosed no abnormalities. ECG illustrated nonspecific repolarization changes. Therefore, the patient underwent surgical excision of the tumor. 

**Figure 1:**
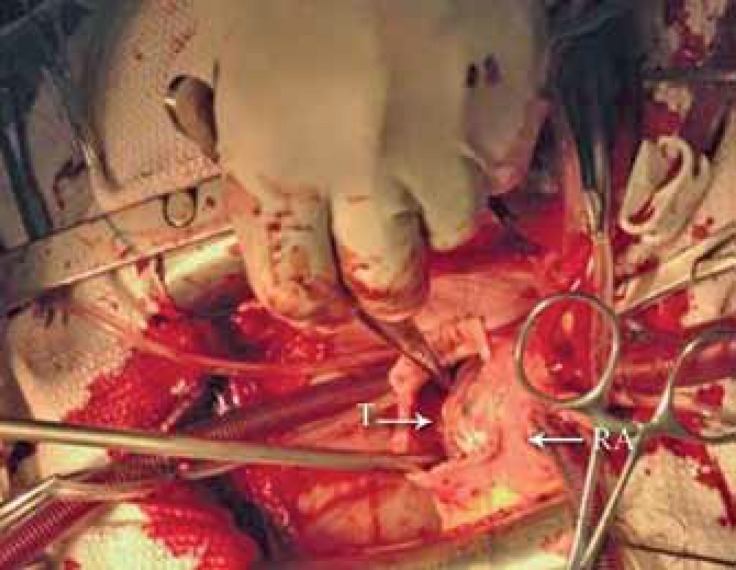
Large tumoral mass in the right atrium.

A huge mass was detected in the right atrium, on the posterior wall, involving the atrioventricular node and the posterior leaflet of the tricuspid valve and protruding into the right ventricular outflow tract. After the radical excision of the tumor, tricuspid valve replacement was done using a biological St. Jude valve (No. 21), and a permanent epicardial pacemaker was established because complete heart block had emerged after radical surgery (Figure 1). The patient’s postoperative period was uneventful. 

**Figure 2:**
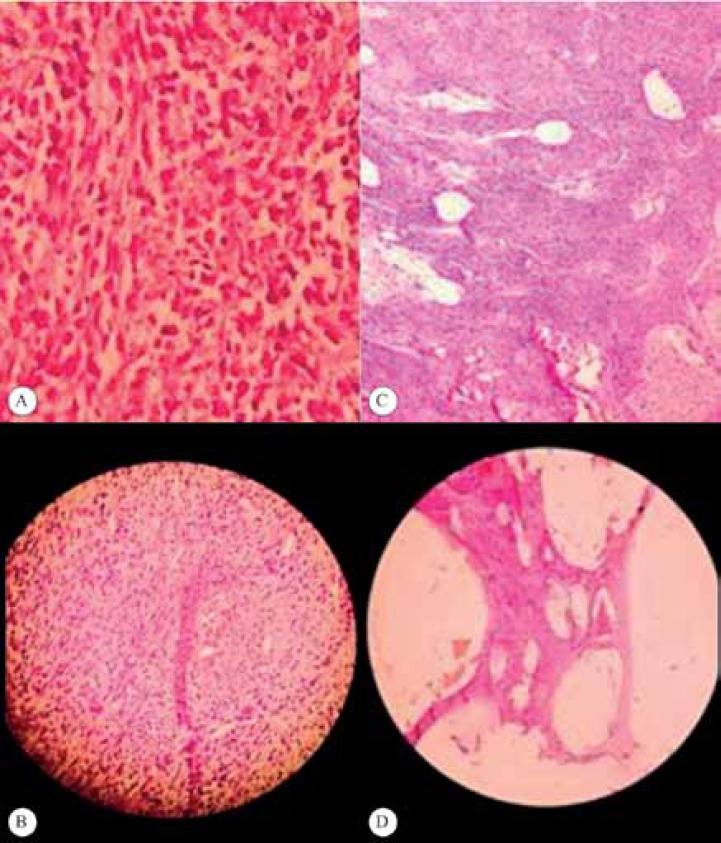
Microscopic view (hematoxylin-eosin staining; magnifications: A and B: × 100, C: × 40, and D: × 10) of the large cardiac tumoral mass (sarcoma). Sections show that the mass is composed of spindle to oval neoplastic cells with a high nuclear-cytoplasmic (N/C) ratio and mitosis. Additionally, there are some foci with a fascicular pattern, necrosis, and hemorrhage.

In the pathological examination, a malignant spindle cell tumor was seen. Desmin and myogenin were negative in the tumoral cells, whereas epithelial membrane antigen, creatine kinase, and vimentin were strongly positive in them (Figure 2). Moreover, morphological and immunohistochemical findings were compatible with a synovial sarcoma.

T, Tumor mass; RA, Right atrium

The patient underwent 3 cycles of adjuvant chemotherapy following radical surgery. At 15 months’ follow-up, she was fortunately having a favorable life quality, and there was no evidence of local or metastatic recurrence in serial TTE and thoracoabdominal CT scan.

## Discussion

Synovial sarcomas usually occur in the soft tissues of the extremities.^3^ Their occurrence in the myocardium is deemed a rare entity. There is a male preponderance of 2.5:1. The right side of the heart is the most frequent location for cardiac synovial sarcomas, but these tumors can occur in the left side of the heart and in the pericardium as well.^5^

Patients with primary synovial sarcomas usually present with dyspnea, chest pain, and evidence of congestive heart failure. These tumors often cause death through widespread infiltration into the myocardium or obstruction of the flow within the heart.^6^ In 33% of the patients, complete surgical resection is possible; however, in those with apparent complete excision, high recurrence rates have been reported.^7^ Treatment is usually palliative resection, followed by chemotherapy with or without radiation.^8^ Bakeen FG et al.^9^ reported the results of the surgical treatment of a series of 27 patients with a median survival of 24 months, and even better results with multi-modality treatment options. Hannachi Sassiet et al.^8^ reported a possible exception to this poor prognosis in the case of a 45-year-old man with a pedunculated right atrial mass, who had complete surgical resection and was reported to be alive for 5 years. In our patient, radical surgery and adjuvant chemotherapy was done, and she was still alive at 15 months' postoperative follow-up without evidence of recurrence. Hopefully, radical surgery combined with chemotherapy and radiotherapy would improve survival in patients with cardiac sarcomas.

Ethical standard: There is no conflict of interests regarding the publication of the present article by any of the writers.

## Conclusion

Due to the rarity of cardiac synovial sarcomas, there are no large studies comparing different therapeutic approaches. Our current case provides further support for the role of radical surgery combined with chemotherapy and radiotherapy.

## Notes:

***This paper should be cited as:*** Hosseinzadeh Maleki M, Aboobakri Makouei M, Hatami F, Zeinabadi Noghabi R. Primary Cardiac Synovial Sarcoma: A Case Report. J Teh Univ Heart Ctr 2017;12(1):32-34.
